# Efficient and Scalable Electrochemical Energy Systems via Peroxide‐Mediated Redox Chemistry

**DOI:** 10.1002/advs.202517218

**Published:** 2025-12-14

**Authors:** Alagar Raja Kottaichamy, Michael Volokh, Jonathan Tzadikov, Menny Shalom

**Affiliations:** ^1^ Department of Chemistry Ben‐Gurion University of the Negev Beer‐Sheva 8410501 Israel; ^2^ Electrochemical Power Sources Division, CSIR‐CECRI Karaikudi Tamil Nadu 630003 India; ^3^ Academy of Scientific and Innovative Research (AcSIR) Ghaziabad 201002 India; ^4^ Ilse Katz Institute for Nanoscale Science and Technology Ben‐Gurion University of the Negev Beer‐Sheva 8410501 Israel

**Keywords:** bifunctional oxygen redox electrocatalysts, hydrogen evolution reaction, oxygen redox reaction, peroxide electrolyzers, peroxide oxidation reaction, rechargeable zinc–air batteries

## Abstract

The transition to renewable energy demands cost‐effective and environmentally sustainable technologies. Electrochemical redox reactions, particularly the oxygen evolution reaction and the oxygen reduction reaction, are central to energy conversion and storage systems such as metal–air batteries, electrolyzers, and fuel cells. However, the conventional four‐electron O_2_ redox pathway suffers from sluggish kinetics and large overpotentials, limiting both efficiency and commercial viability. An emerging alternative is the two‐electron O_2_ redox pathway based on reversible O_2_/H_2_O_2_ conversion. This route offers faster kinetics, lower energy barriers, and a simpler reaction mechanism involving a single intermediate—hydrogen peroxide. This perspective reviews recent progress in two‐electron O_2_ redox chemistry, with an emphasis on its integration into metal–air batteries and water‐splitting systems. Underlying mechanisms, materials challenges, and innovations in catalyst and electrode design that enable efficient, reversible O_2_/H_2_O_2_ cycling are examined. Peroxide‐mediated strategies offer a promising direction for overcoming the limitations of the four‐electron pathway and advancing scalable, high‐efficiency electrochemical energy technologies.

## Graphical abstract

We outline reaction mechanisms and catalyst strategies that enable efficient, reversible O_2_ ⇌ HO_2_^–^ chemistry. Peroxide‐mediated pathways offer an alternative to the conventional 4e^–^/O_2_ route, improving round‐trip energy efficiency in rechargeable zinc–air batteries and supporting scalable hydrogen production without external storage or transportation infrastructure. Key challenges and future opportunities for next‐generation electrochemical energy technologies are also outlined.

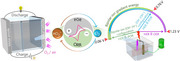

## 1 Introduction

The demand for renewable energy requires scalable, cost‐effective, and eco‐friendly technologies. Central to this goal are electrochemical oxidation and reduction reactions, which drive various energy conversion and storage systems. Among them, the oxygen reduction reaction (ORR) and oxygen evolution reaction (OER) are particularly critical in electrochemical energy devices like rechargeable metal–air batteries, water electrolyzers, fuel cells, and more.^[^ ^1^, ^2^, ^3^, ^4^ ^]^

However, these technologies face performance bottlenecks, partially due to the sluggish kinetics and high overpotentials of the conventional four‐electron (4e^–^) oxygen redox process.^[^ ^5^, ^6^, ^7^, ^8^ ^]^ For instance, while metal–air batteries like the zinc–air battery (ZAB) offer a high theoretical energy density of 1086/1350 Wh kg^−1^ (with/without oxygen), their practical efficiency is constrained by the sluggish 4e^–^ ORR and OER at the air cathode (Equations (1) and (2)).^[^ ^9^, ^10^, ^11^, ^12^, ^13^, ^14^, ^15^ ^]^ Even when employing advanced catalysts,^[^ ^11^, ^12^, ^13^ ^]^ considerable overpotentials contribute to large voltage gaps between charging and discharging, consequently causing round‐trip energy efficiencies (RTEE) to remain below 65%.^[^ ^16^, ^17^, ^18^, ^19^ ^]^

(1) $$\begin{array}{ccc} & & 4e^{-}\;\mathrm{ORR}:O_{2}+4H^{+}+4e^{-}\;\to \;2H_{2}O,E^{\circ }=\\ & & 1.23\;V\;\mathrm{versus}\;\mathrm{reversible}\;\mathrm{hydrogen}\;\mathrm{electrode}\;\left(\mathrm{RHE}\right) \end{array}$$

(2) $$\begin{array}{c} 4e^{-}\;\mathrm{OER}:2H_{2}O\;\to \;O_{2}+4H^{+}+4e^{-},E^{\circ }=1.23\;V\;\mathrm{versus}\;\mathrm{RHE} \end{array}$$

Similarly, water electrolysis, especially in alkaline environments, requires 4.3–5.73 kWh to produce 1 m^3^ of hydrogen, mainly due to high cell voltages (1.8–2.4 V) required to overcome the OER sluggishness.^[^ ^20^, ^21^, ^22^, ^23^ ^]^ This inefficiency persists even with advanced electrode designs, bifunctional catalysts, and engineered electrolytes.^[^ ^24^, ^25^, ^26^, ^27^, ^28^ ^]^ Moreover, both systems often rely on noble metals like Pt and Ir, which drives up costs and limits commercial scalability.^[^ ^29^, ^30^, ^31^ ^]^

One of the most viable ways to overcome these constraints is by replacing the 4e^−^ OER with a faster, lower‐energy alternative. Several approaches have been developed to replace the 4e^–^ OER with an alternative redox system, thus enhancing the operational complexity of the proposed strategy, such as Zn–iodine battery,^[^ ^32^ ^]^ a hybrid Zn‐flow battery,^[^ ^33^ ^]^ and anode‐free ZAB.^[^ ^34^ ^]^ For scalable, energy‐efficient systems, a simpler and more stable aqueous redox couple is needed to bridge the voltage gap between redox reactions. A promising route is the two‐electron (2e^–^) oxygen redox pathway, which produces hydrogen peroxide (H_2_O_2_ or HO_2_^–^ in alkaline media) during ORR. This 2e^–^ pathway offers faster kinetics, reduced overpotentials, and simpler reaction mechanisms compared to the conventional 4e^–^ ORR/OER process. While the 2e^–^ pathway offers theoretical benefits, practical implementation requires further innovation to overcome new bottlenecks, for example, electrocatalyst degradation by the formed hydrogen peroxide.

To this end, we developed a system that couples 2e^–^ ORR and peroxide oxidation reaction (POR) using a nickel‐based bifunctional electrocatalyst.^[^ ^35^ ^]^ This approach leverages the benefits of peroxide redox cycling (O_2_ ⇌ H_2_O_2_/HO_2_^–^), reducing the operational voltage window and improving overall energy efficiency. A major challenge is suppressing the parasitic peroxide reduction reaction (PRR), which degrades H_2_O_2_ into water and oxidizes the electrocatalyst, which undermines the system performance. Therefore, cathode interfaces must be carefully engineered for selective and reversible 2e^–^ redox cycling.

This perspective explores the electrochemical foundation of peroxide‐mediated redox chemistry and its potential to transform ZABs and water‐splitting systems. We contrast the 2e^–^ pathway with traditional 4e^–^ systems and discuss strategies to improve selectivity, longevity, and energy efficiency. Notably, the 2e^–^ peroxide cycle has the potential to push ZAB RTEE above 95%, a significant leap beyond current benchmarks.

In the context of hydrogen production, the peroxide‐based strategy enables on‐site H_2_ generation at nearly half the energy input of conventional water electrolysis.^[^ ^36^ ^]^ By exploiting a bipolar‐ion gradient (OH^–^/H^+^), this system operates at voltages nearly three times lower than standard electrolyzers, achieving high current densities. Estimated energy use is as low as 1.01 kWh per m^3^ H_2_—substantially better than most reported methods.^[^ ^36^ ^]^ This could eliminate the need for expensive hydrogen storage or transportation infrastructure.

## 2 Beyond the 4e^−^ Pathway: A New Peroxide‐Mediated Redox Chemistry

The oxygen redox (ORR and OER), a fundamentally and technologically critical process, plays a central role in the operation of rechargeable air batteries and water‐splitting technologies.

### 2.1 Peroxides in Zinc–Air Batteries

In current ZABs, the cathodic process involves a 4e^−^ ORR during discharge, and the reverse OER during charging, as shown in Equations (1) and (2). Despite their theoretical advantages, both ORR and OER suffer from inherently sluggish kinetics, requiring large overpotentials and often involving the use of complex and expensive electrocatalysts to achieve practical current densities.^[^ ^16^, ^17^, ^18^, ^19^ ^]^ These limitations arise as the 4e^−^ oxygen redox mechanism involves multiple proton‐coupled electron transfer steps through several reaction intermediates, including ^*^OOH, ^*^O, and ^*^OH. The full conversion requires a potential of at least 3.2 eV per 2e^−^, or ≈1.6 V, due to the energy needed to drive transitions between intermediates.^[^ ^11^, ^12^, ^13^, ^37^, ^38^, ^39^ ^]^ Moreover, intrinsic scaling relationships between these intermediates make it difficult to optimize their adsorption energies simultaneously on a single catalyst surface. As a result, materials that efficiently perform ORR are often suboptimal for OER, underscoring the need for bifunctional or dual‐site catalyst designs tailored to each half‐reaction. Real‐time investigation of these multistep intermediates is also difficult. Traditional characterization methods struggle to capture them with the needed temporal and spatial resolution. Nevertheless, a few recent studies have reported significant progress in resolving these steps with improved measurement techniques.^[^ ^40^, ^41^, ^42^ ^]^

Thermodynamically, the minimum required potential is 1.23 V, indicating that even the best catalysts will face an overpotential of ≈0.4 V (≈1.6 V).^[^ ^37^, ^38^, ^39^ ^]^ This overpotential gap limits the maximum RTEE achievable in ZABs. While advances have been made using porous catalysts such as metal–organic frameworks,^[^ ^43^ ^]^ molecular catalysts based on porphyrins and phthalocyanines,^[^ ^44^, ^45^, ^46^ ^]^ and confinement‐effect materials,^[^ ^47^ ^]^ the 4e^−^ O_2_/H_2_O redox couple still suffers from an RTEE of less than 65%—a significant barrier to commercialization.^[^ ^16^, ^17^, ^18^, ^19^ ^]^

To overcome the kinetic limitations of the 4e^−^ pathway, several recent strategies have proposed replacing the traditional OER with alternative redox couples, such as halides or organic mediators.^[^ ^26^, ^27^, ^28^ ^]^ While these systems can deliver higher capacities, they typically require complex zinc redox flow battery architectures, which increase operational complexity and cost.^[^ ^32^, ^33^, ^34^ ^]^ For example, zinc–halide batteries often require sophisticated electrolyte management systems, face reduced long‐term stability, expensive additives, and may emit toxic halogen gases during operation.^[^ ^26^, ^32^, ^33^, ^34^ ^]^

A more stable, efficient, and mainly aqueous redox alternative is needed to maintain the simplicity and compactness of conventional ZAB configurations. One promising approach is the two‐electron oxygen redox pathway, which employs hydrogen peroxide (H_2_O_2_, or HO_2_^–^ in alkaline media) as the oxygen source. Compared to O_2_, H_2_O_2_ offers a more reactive, oxygen‐rich environment and demonstrates significantly faster redox kinetics (in its formation or oxidation) due to its simplified mechanism involving a single intermediate, convenient production and consumption, and a much lower activation barrier.^[^ ^11^, ^12^, ^48^, ^49^, ^50^ ^]^ Additionally, H_2_O_2_ is a liquid, making it more convenient for storage and transport than gaseous or hybrid redox species such as halides.^[^ ^12^, ^51^, ^52^ ^]^ These properties make hydrogen peroxide a particularly attractive candidate for energy storage applications. Consequently, the development and deployment of peroxide‐based ZAB technologies may offer a viable route toward more efficient, safer, and practical energy storage systems in the future.

Two distinct scenarios are conceivable within the two‐electron peroxide redox chemistry framework of rechargeable metal–hydrogen peroxide batteries. In the first scenario, two governing reactions are considered: the peroxide formation reaction (PFR), where H_2_O is oxidized to H_2_O_2_ (Equation (3)), and the PRR, that is, the reduction of H_2_O_2_ to H_2_O (Equation (4)).^[^ ^11^, ^12^ ^]^

a) Peroxide formation and reduction reactions:
(3) $$\begin{array}{ccc} & & 2e^{-}\mathrm{PFR}:2H_{2}O\;\to \;H_{2}O_{2}+2H^{+}+2e^{-},\;E^{\circ }=\\ & & 1.76\;V\mathrm{versus}\mathrm{RHE} \end{array}$$
(4) $$\begin{array}{ccc} & & 2e^{-}\mathrm{PRR}:H_{2}O_{2}+2H^{+}+2e^{-}\to \;2H_{2}O,\;E^{\circ }=\\ & & 1.76\;V\mathrm{versus}\mathrm{RHE} \end{array}$$

**Figure 1:**
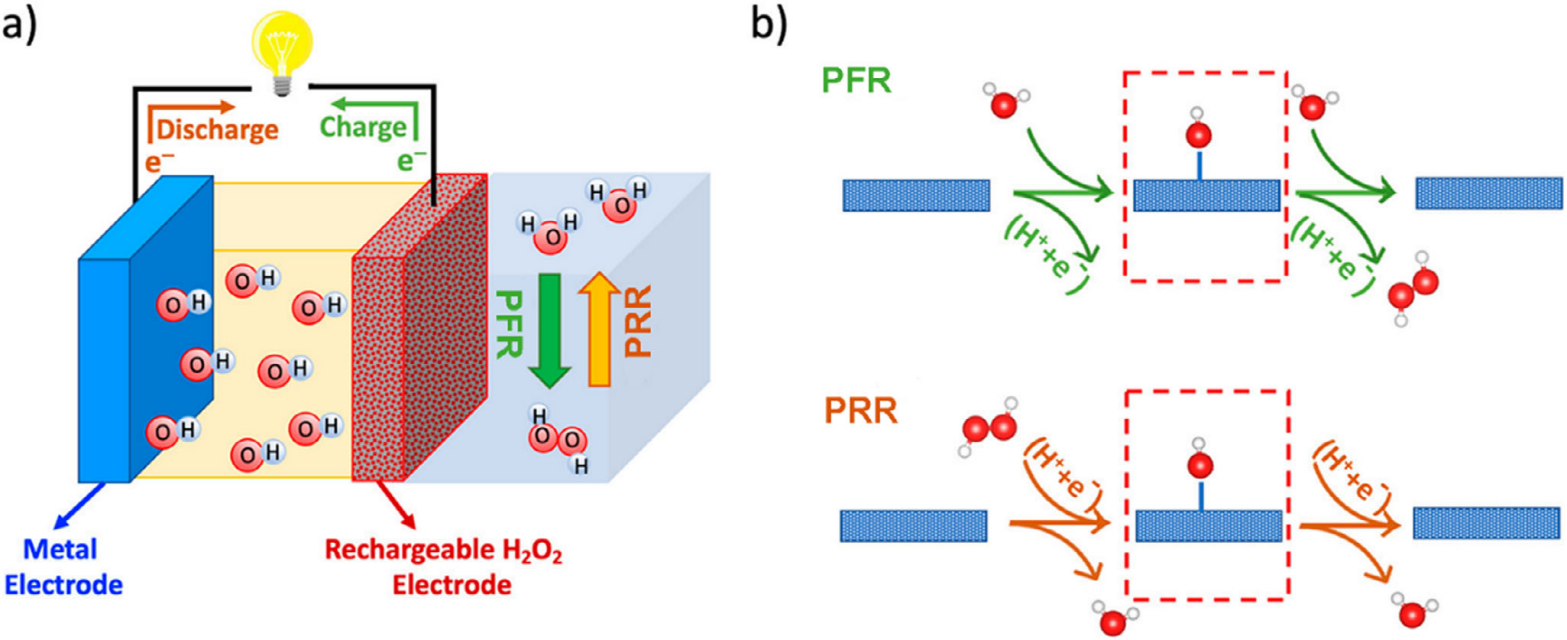
a) Conceptual illustration of the rechargeable metal–H_2_O_2_ battery. b) Reaction pathways of PFR and PRR, dashed squares indicate OH^*^ in both processes. Reproduced with permission from reference,^[^ ^12^ ^]^ copyright 2022, American Chemical Society.

Samira Siahrostami recently highlighted the theoretical potential of exploiting alternative oxygen redox pathways.^[^ ^11^, ^12^ ^]^ In particular, redox processes involving hydrogen peroxide proceed exclusively via 2e^–^ transfer steps, which require smaller overpotentials than the conventional 4e^–^ bifunctional oxygen electrocatalysis.^[^ ^11^, ^12^ ^]^ The proposed rechargeable metal–H_2_O_2_ battery operates through onsite H_2_O_2_ formation (PFR) during charging and H_2_O_2_ reduction (PRR) during discharging (**Figure** **1**a). Both reactions share a single oxygen‐containing intermediate, OH^*^ (Figure 1b), which simplifies the pathway compared to the complex multi‐step oxygen redox in ZABs. This design not only overcomes the intrinsic limitations of oxygen electrocatalysis but also establishes a circular cycle, with H_2_O serving as both the reactant in charging and the product in discharging. The overall efficiency of this system, however, hinges on the development of highly effective PFR and PRR catalysts.^[^ ^11^, ^12^ ^]^ They used computational materials design and thermodynamic analysis to explore catalyst materials, comparing their approach with existing reported catalysts.

The proposed strategy is innovative in regard to eliminating the need for an air cathode and constant air supply, but still poses some challenges, such as the competing OER at the relevant potentials (PFR at ≥ 1.76 V vs RHE), which limits the achievable ZAB energy efficiency.

In contrast to the first scenario, which eliminates the need for O_2_ in the reaction, the second scenario relies on the simple oxidation of hydrogen peroxide (Equation (5)). In this ZAB setup, the charge involves a two‐electron peroxide oxidation reaction (POR), while discharge proceeds through a 2e^–^ORR, resulting in the generation of H_2_O_2_ (Equation (6)).^[^ ^11^, ^48^, ^49^, ^50^ ^]^

b) Two‐electron peroxide oxidation and oxygen reduction reactions:
(5) $$\begin{array}{c} 2e^{-}\mathrm{POR}:\;H_{2}O_{2}\;\to \;O_{2}+2H^{+}+2e^{-},E^{\circ }=0.70\;V\mathrm{versus}\mathrm{RHE} \end{array}$$
(6) $$\begin{array}{c} 2e^{-}\mathrm{ORR}:O_{2}+2H^{+}+2e^{-}\;\;\to \;H_{2}O_{2},E^{\circ }=0.70\;V\mathrm{versus}\mathrm{RHE} \end{array}$$

This pathway offers multiple benefits: 1) POR takes place at lower potentials than the OER, allowing the use of carbon‐based electrodes that would otherwise corrode under OER conditions in alkaline media, thereby reducing reliance on noble metals such as Pt, Ir, and Ru; 2) POR operates with a very small overpotential (≈100 mV), even under high current densities; and 3) neutral electrolytes can be advantageous, providing greater selectivity for H_2_O_2_ production and improved stability during operation, while also creating a less corrosive environment compared to strongly alkaline systems.^[^ ^53^, ^54^ ^]^ **Table** **1** presents a comparison of the 4e^–^ and 2e^–^ O_2_ redox pathways. In summary, the 2e^–^ O_2_ redox pathway has the potential to replace the conventional 4e^–^ pathway in some electrochemical energy devices.

**Table 1:** Comparison of four‐electron and two‐electron oxygen redox chemistry.

System	Reactions	Equations with *E°*
**Four‐electron (4e^–^) O_2_ redox**	Oxygen Reduction Reaction (4e^–^ ORR)	O_2_ + 4H^+^ + 4e^–^ → 2H_2_O; *E°* = 1.23 V vs RHE
	Oxygen Evolution Reaction (4e^–^ OER)	2H_2_O → O_2_ + 4H^+^ + 4e^–^; *E°* = 1.23 V vs RHE
**Two‐electron (2e^–^) O_2_ redox**	Peroxide Reduction Reaction (PRR)	H_2_O_2_ + 2H^+^ + 2e^–^ → 2H_2_O; *E°* = 1.76 V vs RHE
	Peroxide Formation Reaction (PFR)	2H_2_O → H_2_O_2_ + 2H^+^ + 2e^–^; *E*° = 1.76 V vs RHE
	Oxygen Reduction Reaction (2e^–^ ORR)	O_2_ + 2H^+^ + 2e^–^ → H_2_O_2_ (O_2_ + H_2_O + 2e^–^ → HO_2_^–^ + OH^–^, in alkaline media); *E*° = 0.70 V vs RHE
	Peroxide Oxidation Reaction (2e^–^ POR)	H_2_O_2_ → O_2_ + 2H^+^ + 2e^–^ (HO_2_^–^ + OH^–^ → O_2_ + H_2_O + 2e^–^, in alkaline media); *E*° = 0.70 V vs RHE

#### 2.1.1 ZAB Based on Zinc Peroxide Formation

One of the first instances to show the possibility of a peroxide‐based ZAB was shown by Sun W. et al. In this study, a novel reactivity pathway for ZABs operating under neutral pH conditions was introduced, centered on a 2e^–^ O_2_ redox reaction involving the reversible formation of zinc peroxide (ZnO_2_) on carbon‐based cathodes.^[^ ^55^ ^]^ This strategy offers a compelling alternative for achieving highly reversible and sustainable neutral ZAB systems. By suppressing water‐involved side reactions, the approach addresses instability issues in both the zinc anode and the electrolyte, thus enhancing overall battery performance.

**Figure 2:**
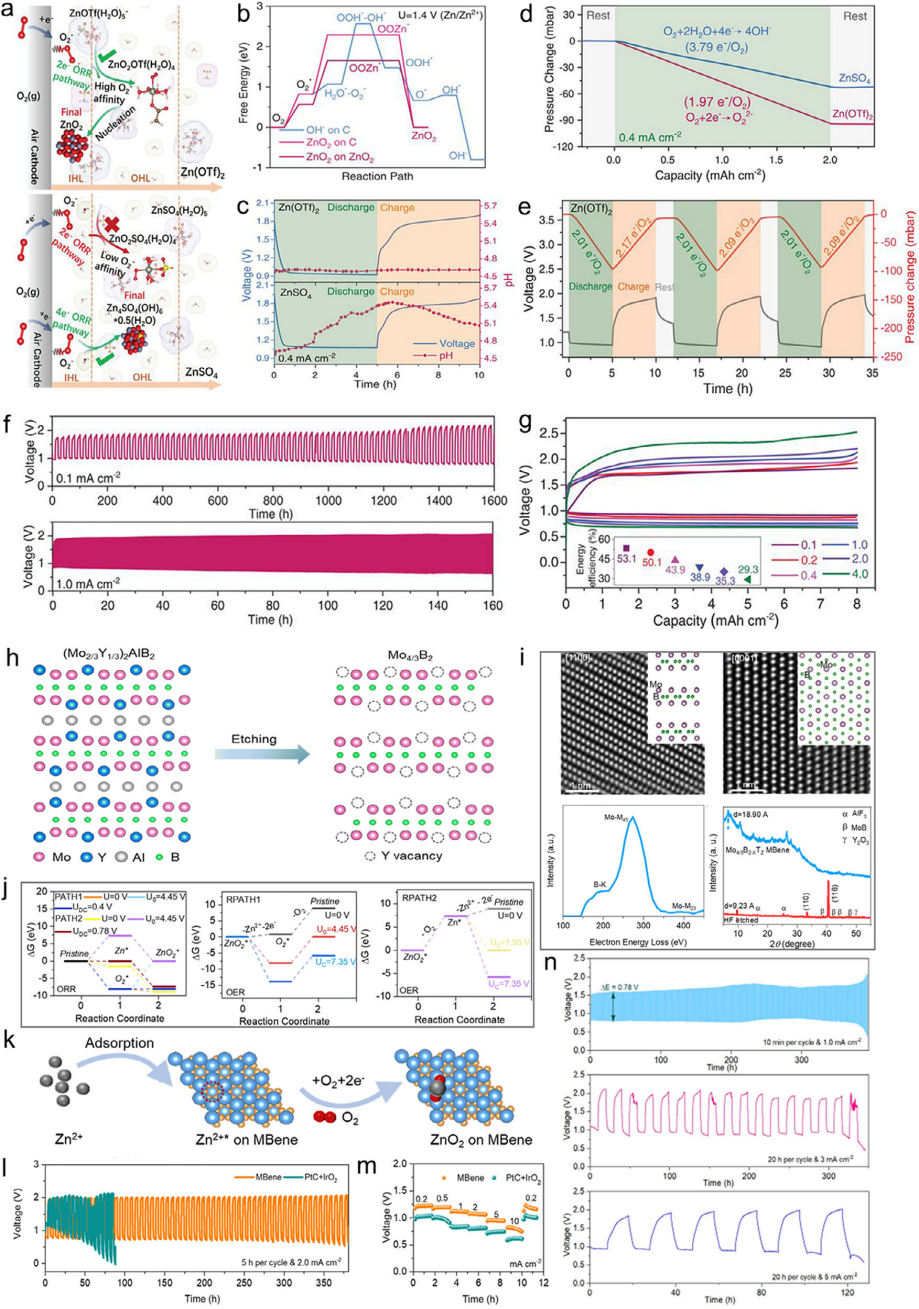
Zn–air batteries utilizing ZnO_2_ chemistry. a) Diagram of interfacial reactions occurring within the IHL and outer Helmholtz layer (OHL) at the air cathode when operating in Zn(OTf)_2_ and ZnSO_4_ electrolytes. b) Calculated free‐energy profiles of ORR pathways: via OH^−^ adsorption on a carbon surface, via ZnO_2_ formation on a carbon surface, and via ZnO_2_ growth on an existing ZnO_2_ surface (*U* = 1.4 V vs Zn/Zn^2+^). c) pH monitoring during galvanostatic discharge, alongside charge/discharge curves of Zn–air cells in Zn(OTf)_2_ and ZnSO_4_ electrolytes at 0.4 mA cm^−2^. d) Gas pressure evolution in Zn–O_2_ cells with Zn(OTf)_2_ and ZnSO_4_ electrolytes during discharge under pure O_2_ atmosphere. e) Pressure variation in Zn–O_2_ cells with Zn(OTf)_2_ electrolyte during galvanostatic cycling at 0.4 mA cm^−2^. f) Cycling stability of Zn–air batteries at current densities of 0.1 and 1 mA cm^−2^. g) Voltage profiles under galvanostatic operation and corresponding RTEE (inset) at a fixed capacity of 8 mAh cm^−2^ across current densities of 0.1, 0.2, 0.4, 1, 2, and 4 mA cm^−2^. Reproduced with permission from reference,^[^ ^55^ ^]^ copyright 2021, Science. Structural analysis, theoretical modeling, and electrochemical behavior of the 2D Mo_4/3_B_2–_ *_x_* T*_z_* MBene cathode. h) Atomic structure of (Mo_2/3_Y_1/3_)_2_AIB_2_ shown before (left) and after etching (right). i) HAADF–STEM images of few‐layer Mo_4/3_B_2–_ *_x_* T*_z_* nanosheets with corresponding atomic configurations (insets), along with electron energy loss spectroscopy (EELS) spectra and XRD patterns of (Mo_2/3_Y_1/3_)_2_AIB_2_ after HF etching (red) and after delamination (blue). j) DFT‐derived energy profiles of the (0001) surface of Mo_4/3_B_2–_ *_x_* T*_z_* for ORR and OER along PATH1/PATH2 and RPATH1/RPATH2. k) Illustration of ORR pathways leading to ZnO_2_ formation on the Mo_4/3_B_2–_ *_x_* T*_z_* surface. l) Cycling stability of near‐neutral ZAB–MBene compared with near‐neutral ZAB–Pt/C + IrO_2_ at 2 mA cm^−2^ under 5 h charge–discharge steps. m) Rate capability of near‐neutral ZAB–MBene versus near‐neutral ZAB–Pt/C + IrO_2_ at different current densities. n) Cycling performance of near‐neutral ZAB–MBene tested at 1, 3, and 5 mA cm^−2^ under both short‐term (10 min) and long‐term (20 h) charge–discharge cycles. Reproduced under the terms of CC‐BY 3.0 license from reference,^[^ ^58^ ^]^ copyright 2023, Royal Society of Chemistry.

To implement this, the researchers designed a near‐neutral electrolyte incorporating strongly hydrophobic anions—specifically, trifluoromethanesulfonate (OTf^–^)—which contain CF_3_ groups. These anions facilitate the formation of a water‐deficient yet Zn^2+^‐rich inner Helmholtz layer (IHL) at the cathode interface, which is additionally supported by free‐energy diagrams. This interfacial configuration enables the reversible precipitation of zinc peroxide (ZnO_2_) on the carbon cathode surface (**Figure** **2**a,b).

A detailed investigation was performed using in situ pH tracking, oxygen pressure measurements during consumption and release, and multi‐cycle tests, interpreted through the ideal gas law and Faraday's law, to clarify the discharge/charge behavior (Figure 2c–e). The Zn–air battery reactions in Zn(OTf)_2_ electrolyte can therefore be described as a reversible ZnO_2_ formation/decomposition process (Zn + O_2_ ⇌ ZnO_2_). Notably, OTf^–^ anions play a critical role in the redox process, being actively involved and partially consumed during ZnO_2_ formation. This leads to improved cycling stability of the ZAB system, Figure 2f. However, despite these improvements, the potential difference between the ORR and OER remains large, similar to other ZAB systems. Still, this work opens pathways for environmentally friendly, air‐stable energy storage systems with fewer side effects than conventional alkaline ZAB chemistries.

However, some challenges using the ZnO_2_‐based chemistry remain. The 2e^–^ ORR in neutral media is kinetically sluggish, resulting in limited current densities.^[^ ^56^, ^57^ ^]^ ZnO_2_ is both insoluble and electrically insulating, hence, blocking the mass transport tunnels and deactivating active catalytic sites, lowering the overall reversible performance. As a result, the RTEE decreased from 53.1% to 29.3% as the current density increased from 0.1 to 0.4 mA cm^−2^ at a fixed capacity of 8 mAh cm^−2^ (Figure 2g). Moreover, at higher current densities, the increased overpotentials favor the competing 4e^–^ OER pathway, which can lead to carbon corrosion at the cathode.

To mitigate these issues, Hou Y. et al. developed an advanced cathode catalyst based on a 2D transition metal boride (MBene), specifically Mo_4/3_B_2–_ *_x_* T*_z_*, incorporating ordered atomic vacancies.^[^ ^58^ ^]^ Both experimental and theoretical analyses showed that *Y*‐site vacancies (arising from etching the (Mo_2/3_ *Y*_1/3_)_2_AlB_2_ precursor) on the MBene surface preferentially adsorb O_2_ over exposed Mo or B sites, although all sites have O_2_ affinity, Figure 2h–j. These vacancies facilitate the reversible 2e^−^ O_2_ redox (ORR/OER), confirming the ZnO_2_ formation–decomposition pathway on the Mo_4/3_B_2–_ *_x_* T*_z_* electrode, shown in Figure 2j–k. Incorporating this MBene catalyst into neutral ZABs yielded significant performance gains. The ZAB–MBene system exhibited long‐term cycling stability exceeding 380 h at 2 mA cm^−2^, surpassing the conventional Pt/C–IrO_2_, **Figure** 2l–m. In addition, the cell maintained decent electrocatalytic activity over varied current densities, with RTEEs of ≈51% at 1 mA cm^−2^ (0.78 V), ≈48% at 3 mA cm^−2^ (1 V), and ≈44% at 5 mA cm^−2^ (1.1 V), Figure 2n. These results underscore the catalyst's robustness in supporting reversible ZnO_2_ chemistry under more demanding conditions.

**Figure 3:**
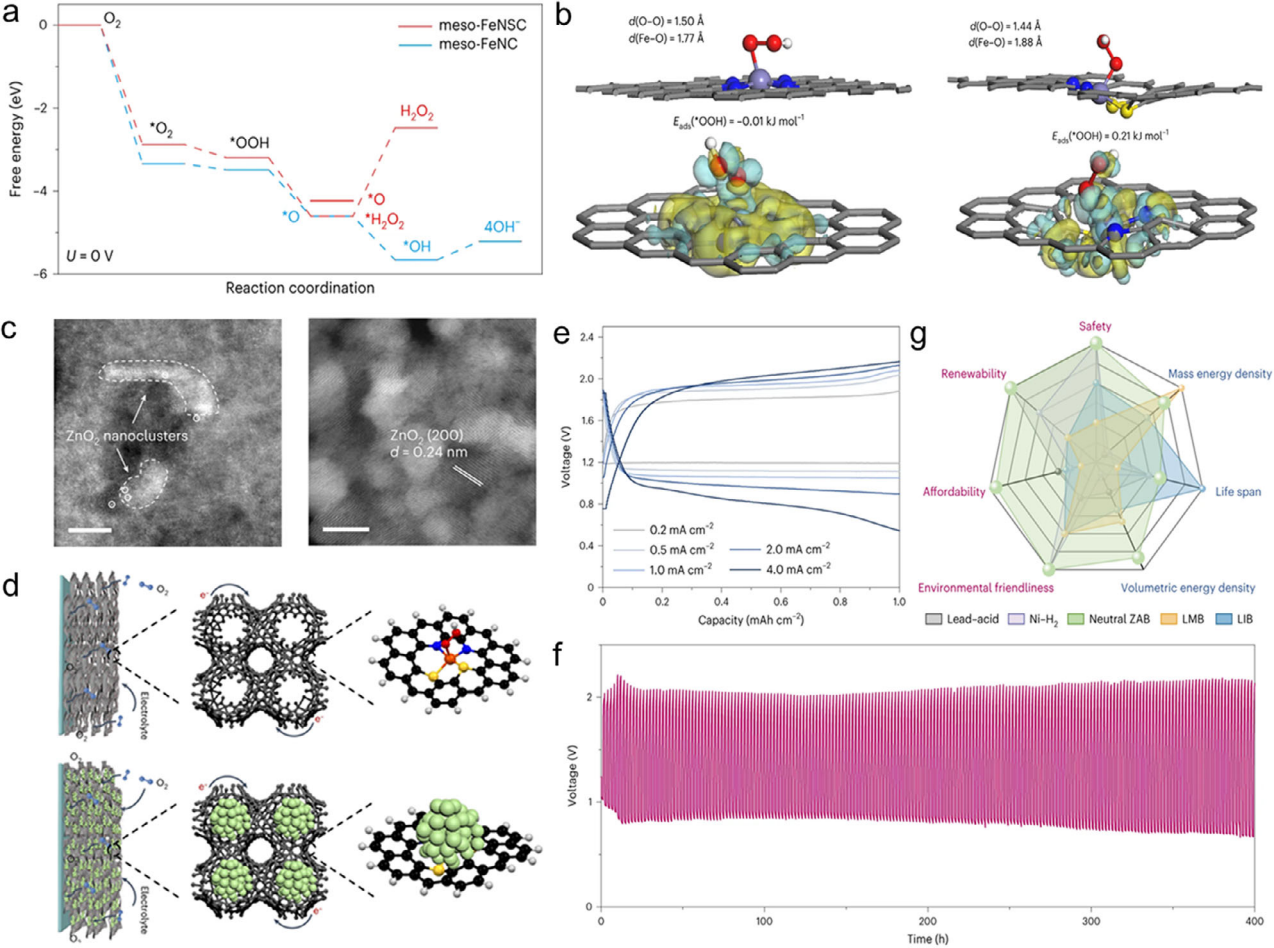
Theoretical analysis, reaction pathways, and electrochemical performance of 2e^–^ neutral ZABs. a) Free‐energy profiles of the ORR on meso‐FeNSC and meso‐FeNC catalysts. b) ^*^OOH adsorption configurations with corresponding charge‐density difference maps for FeN_2_S_2_ and FeN_4_ active sites. The parameters *d* and *E*_ads_ denote the interatomic distance and adsorption energy, respectively. Atom color scheme: C (gray), O (red), H (white), N (blue), S (yellow), and Fe (blue–gray). c) HAADF–STEM and high‐resolution TEM images of meso‐FeNSC discharged to 1 and 0.8 V (scale bars: 5 nm). ZnO_2_ nanoclusters and Fe atoms are indicated with dashed and solid white circles, respectively. d) Schematic illustration of the air‐cathode reaction process in 2e^–^ ZABs assembled with meso‐FeNSC. Color coding: C (black), H (white), Fe (orange), S (blue), N (yellow), and ZnO_2_ clusters (green). e) Galvanostatic discharge/charge profiles of neutral ZABs with meso‐FeNSC at a fixed capacity of 1 mAh cm^−2^ under current densities of 0.2, 0.5, 1, 2, and 4 mA cm^−2^. f) Long‐term cycling durability of neutral ZABs based on meso‐FeNSC at 0.2 mA cm^−2^. g) Performance comparison of 2e^–^ ZABs with other battery systems. Reproduced with permission from reference,^[^ ^59^ ^]^ copyright 2024, Nature.

Expanding on this progress, Wei Zhang and collaborators reported a novel Zn–air battery design incorporating a symmetry‐breaking FeN_2_S_2_ single‐atom catalyst (SAC) supported on mesoporous graphene.^[^ ^59^ ^]^ In this configuration, the asymmetric FeN_2_S_2_ coordination centers preferentially stabilize OOH^*^ intermediates through end‐on adsorption while maintaining the O═O bond. By contrast, symmetric FeN_4_ sites tend to favor OOH^*^ bond cleavage rather than protonation, thus facilitating the direct 4e^–^ ORR, as demonstrated by density functional theory (DFT) calculations (Figure 3a,b). This distinct coordination environment promotes the 2e^–^ ORR pathway, enabling operation at high current densities and supporting reversible ZnO_2_ chemistry. Their stepwise synthesis strategy—combining pore engineering, graphitization, and atomic‐level coordination control—successfully addressed the typical compromise between catalytic activity and structural stability in SACs. Typically, increasing graphitization boosts stability at the expense of catalytic accessibility, while higher porosity boosts activity but compromises structure. By combining both, the resulting mesoporous graphene offers a high specific surface area and fast mass/e^–^ transport, creating an ideal nanoscale reactor to confine ≈6 nm ZnO_2_ discharge products, and this spatial confinement ensures full reversibility of the ZnO_2_ reaction without generating unwanted byproducts, as illustrated in Figure 3c,d.

The asymmetrical FeN_2_S_2_ sites exhibit structural flexibility, enabling dynamic rearrangements that enhance oxo‐species adsorption. This adaptability supports efficient gas–liquid–solid interactions, which are critical for driving selective 2e^–^ pathways and achieving high‐rate performance in ZABs, as shown in **Figure** 3e. The system delivered a RTEE of 61% at 0.2 mA cm^−2^ and demonstrated stable performance for more than 400 h (Figure 3f). These results surpass all previously reported neutral ZABs. When compared with other 2e^–^ neutral ZABs and mainstream battery technologies such as lithium‐ion batteries, lithium metal batteries, lead–acid, or nickel‐based batteries, this system offers notable advantages in safety, renewability, affordability, and environmental friendliness—underscoring its strong potential as a next‐generation energy storage solution (Figure 3g).

**Figure 4:**
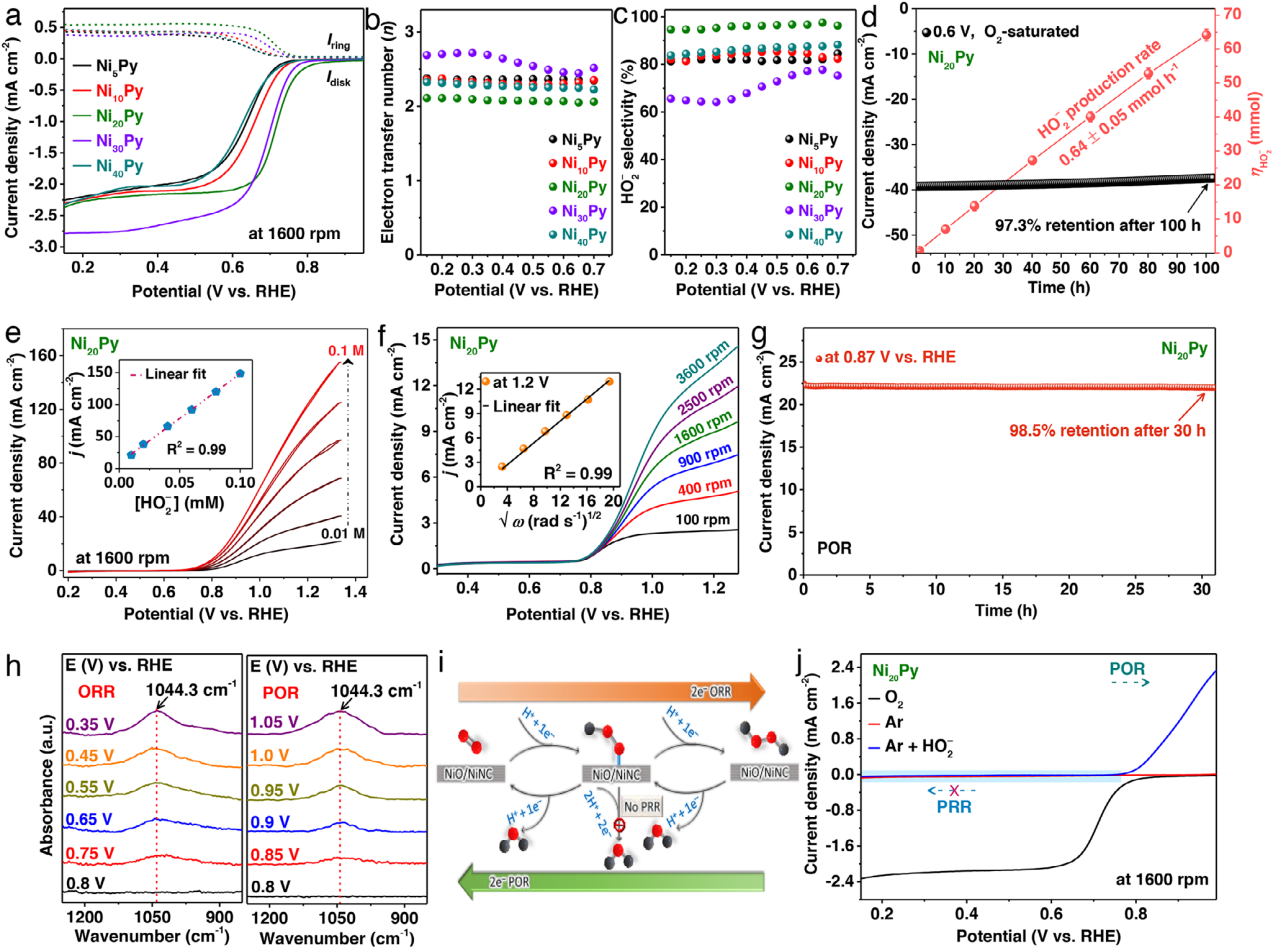
Evaluation of ORR/POR electrocatalysis and interfacial examinations. a) ORR activity of the Ni*_x_* Py catalyst in O_2_‐saturated 0.5 m KOH, showing disk currents at 1600 rpm along with ring currents from peroxide formation. b) Calculated apparent electron transfer number (*n*). c) HO_2_^–^ selectivity (%). d) Durability test: current density and HO_2_^–^ yield monitored over time at 0.6 V versus RHE in an H‐cell configuration. The error bars indicate the standard deviation of experimental data. e) Current responses at HO_2_^–^ concentrations ranging from 0.01 to 0.1 m at 1600 rpm in 0.5 m KOH; inset: plot of peak current density (*j*) versus [HO_2_^–^]. f) Rotating disk electrode measurements of a Ni_20_Py‐modified glassy carbon electrode; inset: corresponding Levich plot at 1.2 V versus RHE. g) POR stability curve (*j–t*) for Ni_20_Py at 0.87 V versus RHE in 0.5 m KOH containing 0.1 m HO_2_^–^. h) In situ ATR–FTIR spectra of Ni_20_Py recorded under different applied potentials during ORR and POR. i) Schematic of the reversible ORR/POR mechanism. j) Current density curves of the 2e^–^ ORR and POR in O_2_‐ and Ar‐saturated 0.5 m KOH with 2 mm HO_2_^–^ at 10 mV s^−1^ and 1600 rpm, showing suppression of the peroxide reduction reaction (PRR). The Ni_20_Py catalyst enables HO_2_^–^ production over a broad potential window (0.15–0.79 V vs RHE). Reproduced under the terms of the CC‐BY license from reference,^[^ ^35^ ^]^ copyright 2024, published by Wiley–VCH.

Despite these advances, significant challenges persist. The 2e^–^ ORR in neutral media continues to suffer from intrinsically slow kinetics and elevated overpotentials, which reduce the RTEE, especially under practical operating current densities. Additionally, the insoluble and electrically insulating nature of ZnO_2_ leads to blockage of mass transport channels and progressive deactivation of catalytically active sites. Even under well‐optimized conditions, the RTEE seldom surpasses 61% within the 0.1–4 mA cm^−^^2^ (1–8 mAh cm^−^^2^) range,^[^ ^55^, ^58^, ^59^ ^]^ primarily because of the energy cost linked to ZnO_2_ redissolving and Zn–O bond cleavage.

At higher current densities, the system often shifts toward the competitive 4e^−^ OER pathway, driven by increasing overpotentials.^[^ ^55^, ^58^, ^59^ ^]^ Although this pathway is more kinetically favorable, it undermines the selectivity and reversibility of the ZnO_2_‐based chemistry. This trade‐off underscores a core limitation: while the 2e^–^ mechanism offers theoretical advantages in terms of energy efficiency and environmental sustainability, its practical realization demands transformative progress in catalyst engineering and reaction environment control. Overcoming these bottlenecks remains essential for unlocking the full potential of ZABs as next‐generation sustainable energy storage systems.

#### 2.1.2 ZAB Based on Peroxide‐Mediated Redox Chemistry

**Scheme 1:**
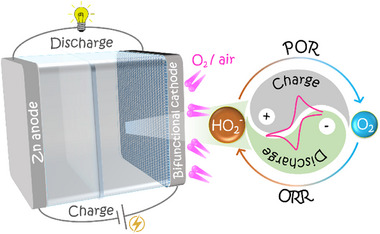
Schematic representation for a two‐electrode configuration of an alkaline zinc–peroxide battery (ZPB) based on an oxygen 2e^–^ redox process. POR stands for the 2e^–^ peroxide (i.e., HO_2_^–^) oxidation reaction, ORR for the 2e^–^ oxygen reduction reaction at the bifunctional cathode.

In response to the challenges facing ZABs, in our recent study, we have explored a new approach in alkaline ZAB configuration based on the onsite formation and oxidation of HO_2_^–^ at a bifunctional air cathode (**Scheme** **1**).^[^ ^35^ ^]^ This system, henceforth referred to as a Zn–peroxide battery (ZPB), leverages a 2e^–^ oxygen redox pathway wherein the discharge process involves the ORR and the generation of HO_2_^–^ on the air cathode (Equation (6)). During charging, the same HO_2_^–^ is oxidized back to O_2_ and H_2_O through the 2e^–^ POR (Equation (5)), Scheme 1.

A key requirement of this strategy is the design of a bifunctional catalyst that delivers both high activity and durability for ORR and POR. We have designed and synthesized an efficient, cost‐effective, and stable bifunctional electrocatalyst comprising NiN*_x_* C*_y_* single‐atom active sites and Ni‐based nanoparticles (Ni(OH)_2_), embedded in a crystalline carbon matrix. This design builds upon our previous reports.^[^ ^60^, ^61^ ^]^

The resulting catalyst, referred to as Ni_20_Py, demonstrates state‐of‐the‐art performance with ORR selectivity of 96% ± 2% within a voltage window of 0.15–0.79 V versus RHE and measured peroxide production rate reaches ≈1574 mmol g_cat_^−1^ h^−1^ at 0.4 V versus RHE, with sustained stability over 100 hours for peroxide generation. Supporting data, including concentration profiles, Levich plots, and durability tests for peroxide oxidation in alkaline conditions, suggest its potential bifunctional activity (Figure 4a–g). Furthermore, the calculated energy cost for HO_2_^–^ production on Ni_20_Py via the 2e^–^ ORR pathway is 152 kJ mol^−1^, which is markedly lower than those of previously reported catalysts.^[^ ^35^, ^62^ ^]^

In situ attenuated total reflectance–Fourier‐transform infrared (ATR–FTIR) spectroscopy across various potentials, coupled with theoretical modeling, confirms that the bifunctional catalyst stabilizes an adsorbed hydroperoxyl intermediate (OOH)^*^, Figure 4h,i. This intermediate is critical for both the ORR and POR, facilitating the seamless reversibility of the hydroperoxide chemistry. Importantly, the Ni_20_Py catalyst exhibits minimal PRR, the undesired conversion of HO_2_^–^ to OH^–^, over a broad potential window (0.15–0.79 V), as illustrated in Figure 4j. This suppression of parasitic pathways ensures high selectivity and supports efficient bifunctional performance in the ZPB architecture, Scheme 1.

**Figure 5:**
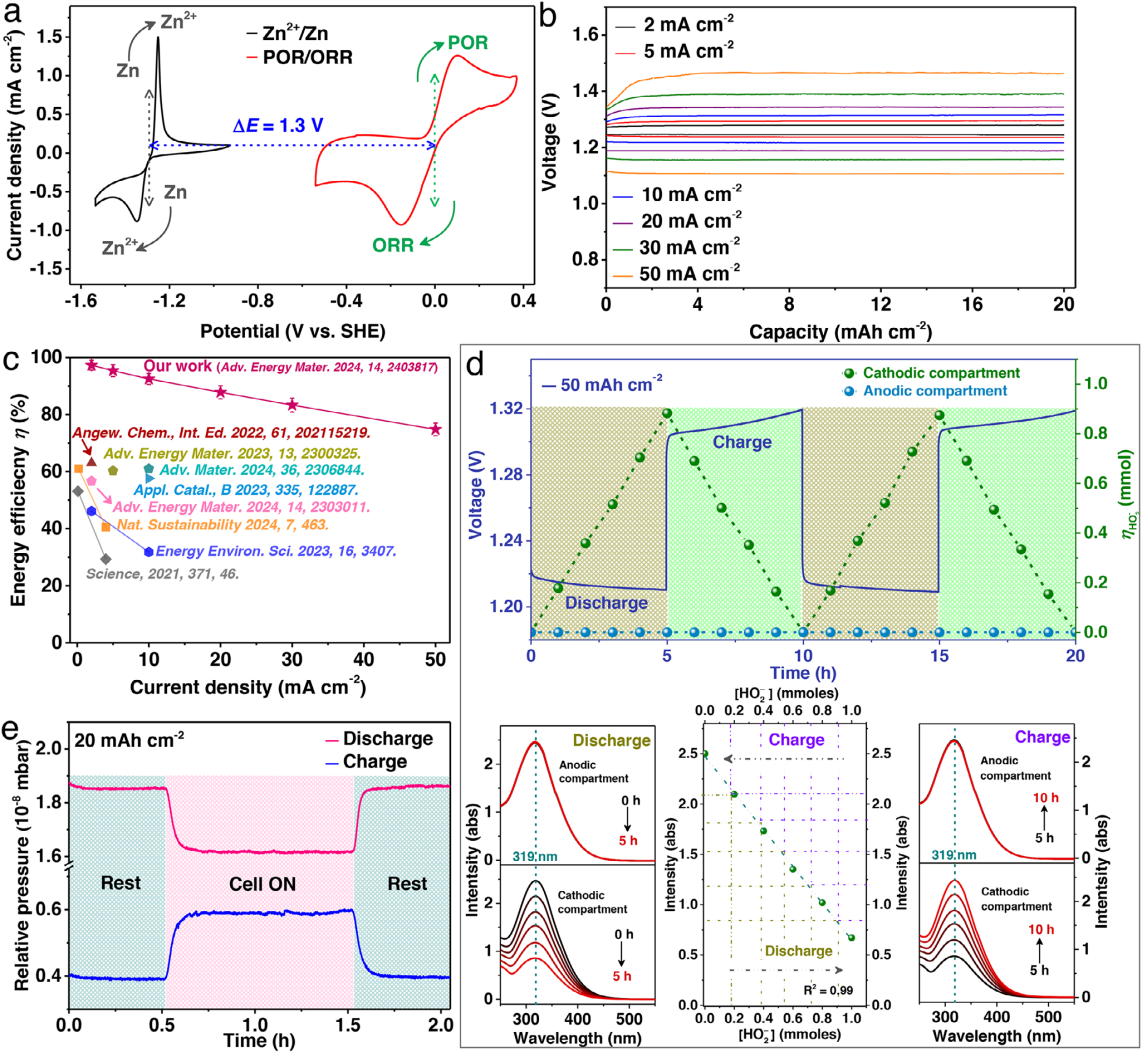
Performance of the rechargeable ZPB. a) Cyclic voltammograms of the deposition and stripping of Zn in an alkaline medium and of the 2e^–^ POR and ORR on a Ni_20_Py. b) Galvanostatic charge–discharge rate profiles of ZPB in a capacity fixed mode (20 mAh cm^−2^) at increasing current densities (mA cm^−2^). c) Plot of energy efficiency (𝜂) versus current density (j) of the proposed ZPB compared with representative ZAB reports. d) Measuring changes in HO_2_^–^ concentration in the ZPB configuration. Discharge–charge profile at a rate of 50 mAh cm^−2^ (blue line) and the accompanying changes in HO_2_^–^ concentration, shown dashed lines (the green dashed line represents the cathode compartment, while the cyan dashed line represents the anode compartment). Monitoring changes in HO_2_^–^ concentration in anode/cathode compartments during ZPB operation (charge/discharge) and respective solutions added to Ce(SO_4_)_2_ titrant solutions or UV–vis spectroscopy at different time intervals. e) Pressure change of ZPB monitored via in situ electrochemical mass spectrometry during galvanostatic discharge and charge at a constant capacity of 20 mAh cm^−2^. Reproduced under the terms of the CC‐BY license from reference,^[^ ^35^ ^]^ copyright 2024, published by Wiley–VCH.

A voltage difference of ≈1.3 V is observed when comparing the redox features of Zn/Zn^2+^ (black trace) and O_2_/HO_2_^–^ (red trace) in Ni_20_Py catalyst half‐cells (**Figure** **5**a). Integration of the Ni_20_Py bifunctional catalyst into the ZPB system delivers an exceptionally low initial charge potential, 1.28 V at 2 mA cm^−2^ (20 mAh cm^−2^ fixed capacity), and maintains only 1.48 V even under a high current density of 50 mA cm^−2^ (Figure 5b). These performance metrics correspond to RTEEs of 97.3% and 74.8%, respectively. The minimal potential gap of only 60 mV between the 2e^–^ ORR/POR highlights the fast kinetics of both processes, marking a paradigm shift in peroxide‐mediated systems by dramatically improving round‐trip energy efficiency and reducing charging voltages over the existing ZABs, as illustrated in Figure 5c.

The dynamic formation and consumption of HO_2_^–^ during ZPB operation were confirmed using UV–vis spectroscopy to monitor changes in HO_2_^–^ concentration in the electrolyte (Figure 5d). A controlled charge–discharge experiment was performed at a capacity of 50 mAh cm^−2^ (5‐hour charge and discharge per cycle). As shown in Figure 5d, HO_2_^–^ concentration (green dashed line) increased steadily during discharge, consistent with the two‐electron ORR pathway, and declined during charging as HO_2_^–^ was consumed via POR. Notably, throughout all measurements, no significant crossover of HO_2_^–^ to the anodic compartment was detected (Figure 5d, blue dashed line), affirming effective ion confinement. In situ electrochemical mass spectrometry further validated the electrochemical reversibility of the system (Figure 5e). During discharge at a constant capacity of 20 mAh cm^−2^, the partial pressure of O_2_ (pink trace) decreased as it was consumed in the ORR. During charging, the O_2_ partial pressure (blue trace) increased accordingly, reflecting oxygen evolution from the POR, consistent with the closed‐loop operation of the ZPB.

**Figure 6:**
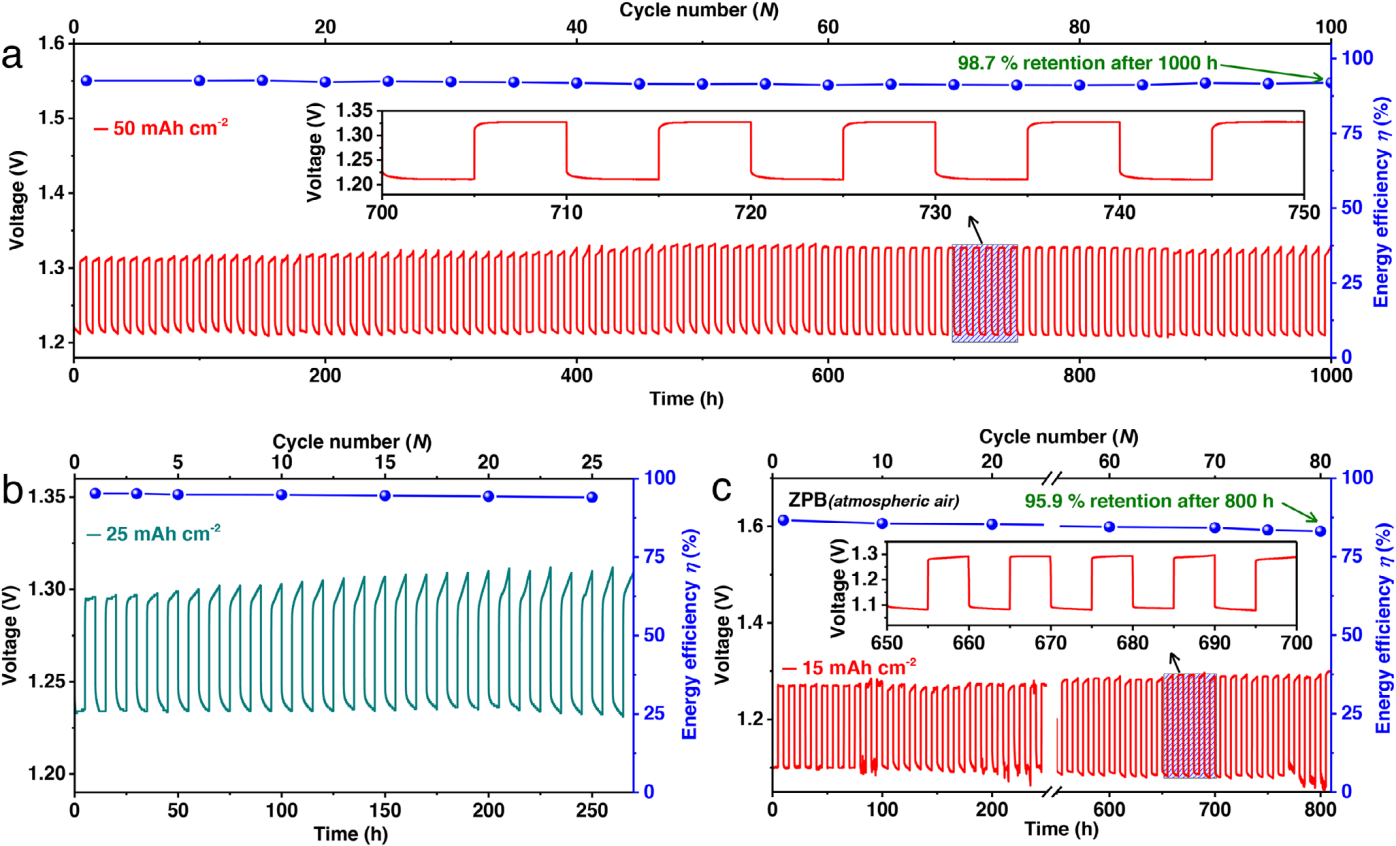
Durability study of the rechargeable ZPB. Cycling performance of two‐electrode Zn–O_2_ batteries using Ni_20_Py as the catalyst of a bifunctional air electrode at a capacity of a) 50 mAh cm^−2^ (*j *= 10 mA cm^−2^) and b) 25 mAh cm^−2^ (*j *= 5 mA cm^−2^) in a sealed battery setup with a sufficient amount of oxygen (≈0.5 mmol of O_2_) using 10 h charge–discharge per cycle; the energy efficiency corresponds to the ratio between the charge and discharge voltage plateaus (the magnified inset in Figure 6a shows the data between 700 and 750 h). c) Cycling performance of the ZPB where the oxygen source is atmospheric air, at a fixed capacity of 15 mAh cm^−2^ (*j *= 3 mA cm^−2^) (the magnified inset in Figure 6c shows the data between 650 and 700 h). Reproduced under the terms of the CC‐BY license from reference,^[^ ^35^ ^]^, copyright 2024, published by Wiley–VCH.

The ZPB's practical applicability was evaluated under three different operating modes (**Figure** **6**):

- O_2_(g) flow mode: When supplied with a continuous stream of oxygen gas, the ZPB cell successfully completed 100 charge–discharge cycles, corresponding to ≈1000 h of operation, at an areal capacity of 50 mAh cm^−2^ and a current density of 10 mA cm^−2^ (Figure 6a). Throughout this process, the system delivered an average energy efficiency of ≈92% and preserved 98.7% of its initial efficiency after 100 cycles.
- Sealed cell mode: In a sealed‐cell configuration, where 0.5 mmol of O_2_ was preloaded into the air electrode chamber (Figure 6b), the battery consistently provided a stable capacity of 25 mAh cm^−2^ at 5 mA cm^−2^. Remarkably, the device maintained an energy efficiency of ≈95% throughout operation.
- Open‐air mode: Operating directly in ambient air without external oxygen feeding, the ZPB cell functioned at a fixed capacity of 15 mAh cm^−2^ and a current density of 3 mA cm^−2^. This setup showed excellent durability and reversibility, sustaining ≈83% efficiency during long‐term cycling and retaining ≈96% of its initial efficiency even after more than 800 hours of continuous operation (Figure 6c).

In summary, the 2e^–^ peroxide‐redox chemistry as realized, for example, with the developed Ni‐based bifunctional air cathode, enables rapid formation and consumption of peroxide, high energy efficiency, long durability, and high capacity. This new cathode chemistry represents a meaningful step toward advancing high‐energy‐density metal–air batteries.

### 2.2 Peroxides in Alkaline Water Electrolyzers

**Figure 7:**
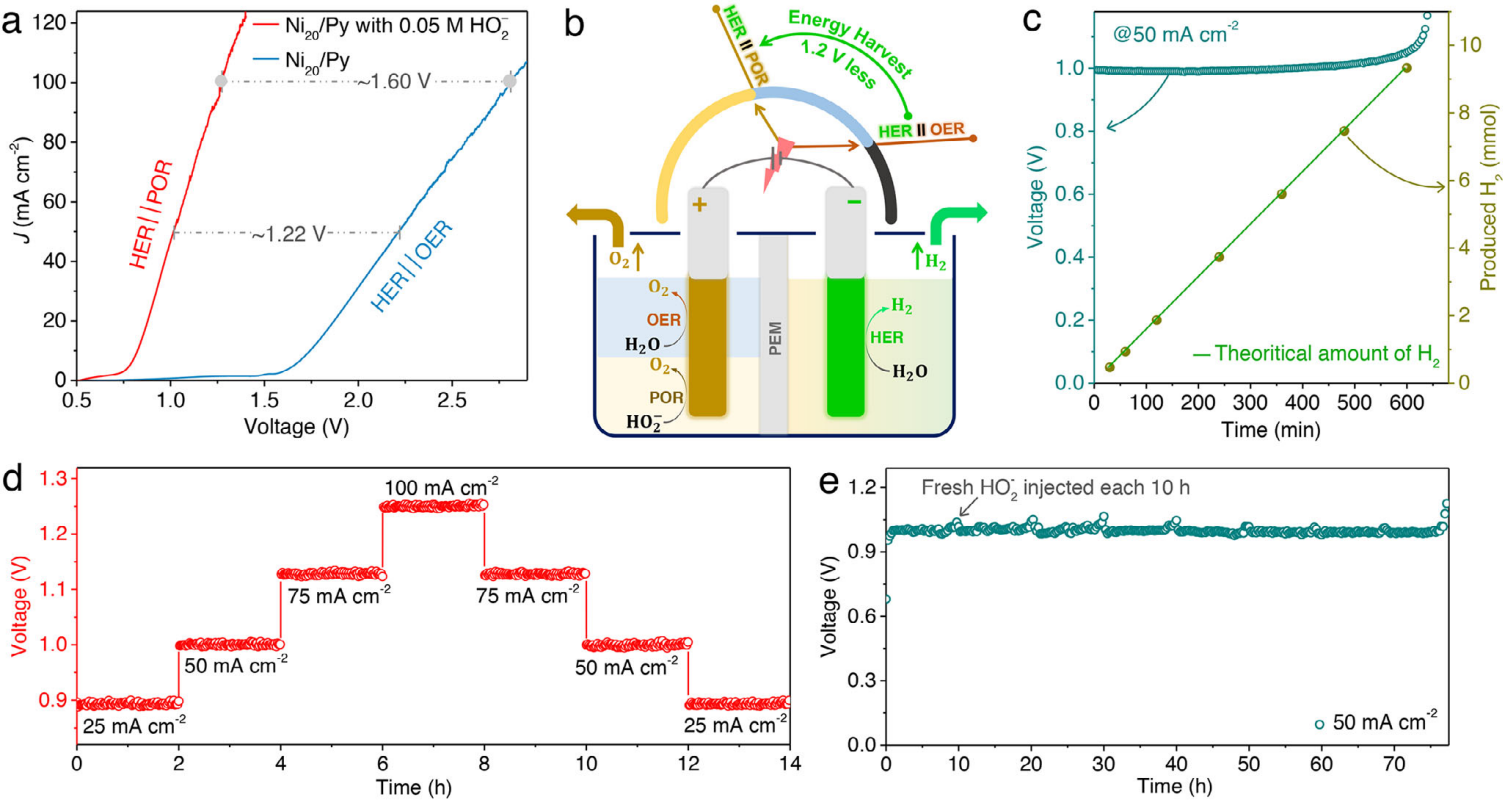
Alkaline peroxide electrolyzer (APE) performance. a) *J–E* profiles comparing APE (driven by POR) with conventional AE (driven by OER). b) Schematic illustration of the dual‐compartment setup for both APE and AE. c) Hydrogen evolution in the cathodic chamber along with the corresponding *E–t* trace, where the green line denotes the theoretical H_2_ output. d) Rate characteristics of the APE cell measured at different applied current densities (*J*, mA cm^−2^). e) Long‐term stability of the APE employing the Ni_20_Py catalyst at a constant current density of 50 mA cm^−2^ in 2 m KOH, with fresh 0.2 m HO_2_^–^ replenished every 10 h. Reproduced under the terms of the CC‐BY license from reference,^[36]^ copyright 2024, published by Wiley–VCH.

**Scheme 2:**
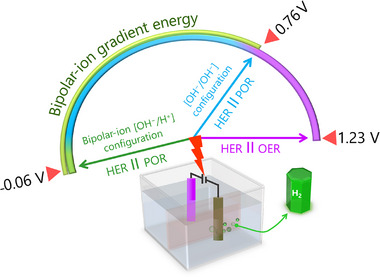
Schematic representations of an electricity‐efficient peroxide electrolyzer for H_2_ fuel production. Reproduced under the terms of the CC‐BY license from the reference, copyright 2024, published by Wiley–VCH.

In the context of alkaline water electrolysis (AWE), most efforts are focused on improving electrocatalyst activity in OER^[^ ^63^ ^]^ and developing a non‐platinum‐group metal (PGM) cathode (where HER occurs) electrocatalyst.^[^ ^64^ ^]^ Still to this day, the oxygen evolution reaction (OER) at the anode is the main bottleneck due to its slow kinetics and high overpotential requirements. This results in high overall cell voltages of 2–2.8 V, making AWE operation energetically high. However, examining the potentials of POR (*E°* = –0.08 V vs standard hydrogen electrode (SHE) in alkaline media) and HER (*E°* *= *–0.83 V vs SHE in alkaline media) could potentially result in a cell voltage of ≈0.75 V, greatly decreasing the energy requirement of AWE. One of the most used strategies to overcome OER limitations is replacing it with faster oxidation reactions, while acquiring added‐value chemicals. These reactions include the oxidation glycerol,^[^ ^65^ ^]^ hydrazine,^[^ ^66^ ^]^ methanol,^[^ ^67^ ^]^ glucose,^[^ ^68^ ^]^ and more.^[^ ^69^ ^]^ For that matter, we replaced it with the faster POR in a recent study conducted in our group. The rapid kinetics of POR allow hydrogen to be produced at significantly lower voltages compared to OER (**Figure** **7**a,b).^[^ ^48^, ^49^, ^50^ ^]^ This approach provides key benefits for sustainable hydrogen generation, storage, and delivery. Using an inexpensive, efficient, and durable bifunctional catalyst for hydrogen peroxide, we showcase three electrolyzer designs capable of lowering the theoretical cell voltage from the standard 1.23 V to as little as –0.06 V (**Scheme** **2**).^[^ ^36^ ^]^

In every configuration, the hydrogen is produced at the cathode through an HER, while hydrogen peroxide undergoes oxidation at the anode via the POR to yield oxygen. Aside from reducing the overall energy requirement, peroxide also functions as a practical hydrogen carrier. Importantly, it can be synthesized and replenished on‐site using renewable electricity, establishing a self‐sustaining, closed‐loop pathway for localized hydrogen delivery. This regenerative cycle addresses major drawbacks of other hybrid electrolyzer systems, such as those relying on hydrazine or alcohols, that demand far greater energy consumption.^[^ ^70^, ^71^, ^72^ ^]^

#### 2.2.1 Alkaline Peroxide Electrolyzer (APE)

We constructed an alkaline peroxide electrolyzer using a zero‐gap configuration.^[^ ^36^ ^]^ At a current density of 100 mA cm^−2^ (Figure 7a), the APE requires a cell voltage of ≈1.26 V, compared to ≈2.8 V for a conventional AWE, and 1.6–2 V in anion exchange water electrolyzers. This ≈1.54 V reduction highlights the opportunities of employing the POR over the OER at the anode (Figure 7b).

Hydrogen production from the APE prototype was assessed at a current density of 50 mA cm^−2^ in 2 m KOH containing 0.2 m HO_2_^–^, using the water displacement technique (Figure 7c). The generated hydrogen reached ≈9.2 mmol cm^−2^, in excellent agreement with the theoretical values across all intervals. This corresponds to a Faradaic efficiency of 98.5%. To test durability, the device was subjected to variable current cycling, shifting between 25 and 100 mA cm^−2^ before returning to 25 mA cm^−2^. In all cases, the initial performance was recovered without significant voltage drift (Figure 7d), indicating stable catalytic activity. Durability testing over 75 h at ≈1 V demonstrated the robustness of the Ni_20_Py catalyst in highly oxidative conditions (Figure 7e). Overall, replacing the slow OER with the fast POR in the anodic compartment enables substantially lower operating voltages and stable long‐term performance, making APE a promising platform for efficient hydrogen production.

#### 2.2.2 On‐Site Regenerative Peroxide‐Mediated APE

**Figure 8:**
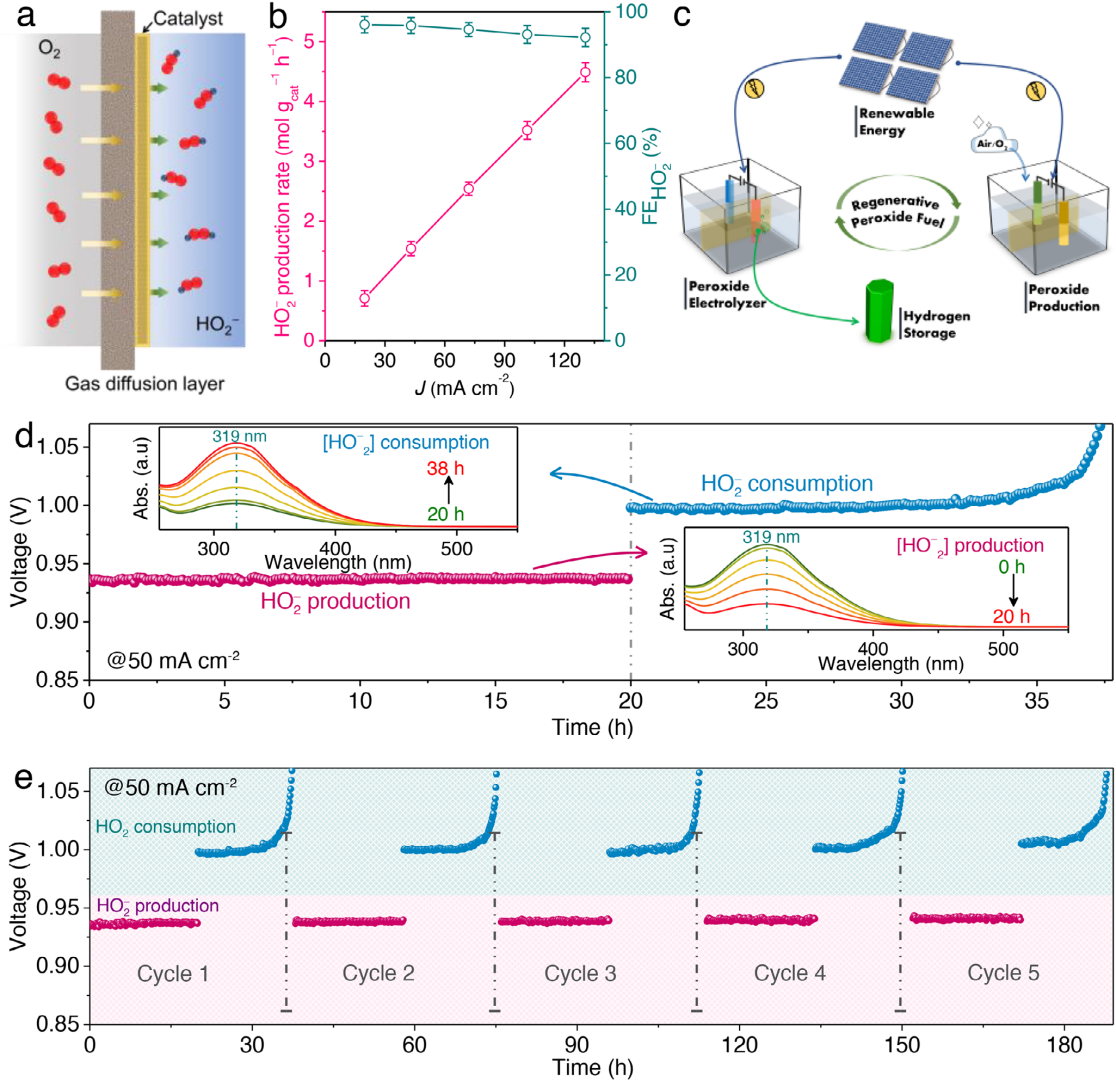
Evaluation of peroxide generation and regenerative fuel cycling. a) Diagram of a flow‐cell setup illustrating HO_2_^–^ formation at the GDL electrode interface (electrolyte/catalyst/O_2_ boundary). b) HO_2_^–^ generation rates and Faradaic efficiencies obtained with a Ni_20_Py‐modified GDL electrode across various current densities; the HO_2_^–^ FE is determined from the ORR process. c) Conceptual schematic of regenerative HO_2_^–^ fuel production enabled by the bifunctional Ni_20_Py catalyst. d) Regenerative cycling profile of HO_2_^–^ at 50 mA cm^−2^, with the inset showing UV–vis monitoring of HO_2_^–^ concentration over time during production and consumption by titration with cerium sulfate solutions. e) Repeated regenerative HO_2_^–^ production–consumption cycles at a constant current density of 50 mA cm^−2^. Reproduced under the terms of the CC‐BY license from reference,^[^ ^36^ ^]^ copyright 2024, published by Wiley–VCH.

HO_2_^–^ was produced at the three‐phase interface formed by the electrolyte, gaseous O_2_, and the solid electrocatalyst deposited on a gas diffusion layer (GDL), specifically Ni_20_Py@GDL (**Figure** **8**a), in a commercial‐type configuration. At 0.3 V versus RHE and 130 mA cm^−2^, the HO_2_^–^ generation rate has reached 4.5 ± 0.2 mol g_cat_^−1^ h^−1^ (Figure 8b), showing a clear linear increase with the applied current. These findings demonstrate that Ni_20_Py can operate at high current densities while facilitating POR and ORR, acting as a bifunctional electrocatalyst. This enables the possibility to decouple water‐splitting into two steps: i) on‐the‐spot HO_2_^–^ synthesis in electrolysis mode, and ii) on‐demand H_2_ generation without relying on complicated hydrogen transport infrastructure. In this system, HO_2_^–^ functions as the fuel for the APE, supporting a regenerative cycle where fuel is replenished directly over the Ni_20_Py catalyst, thereby removing the need for external hydrogen peroxide delivery (Figure 8c).

A proof‐of‐concept study validated the feasibility of cycling between HO_2_^–^ generation and subsequent utilization for hydrogen production. Real‐time UV–vis colorimetric monitoring confirmed the stepwise production and consumption of HO_2_^–^ in separate compartments during the regenerative operation (Figure 8d, inset). In the initial 20 h cycle, 16.63 mmol of HO_2_^–^ was obtained, corresponding to a Faradaic efficiency of 89.12% (Figure 8d). When the counter electrode was switched from Ni foam to Pt mesh, the accumulated HO_2_^–^ was consumed in the following stage, yielding ≈16.05 mmol over 17.8 h with a Faradaic efficiency of 96.68%, giving a total operation time of 37.8 h (Figure 8d). While at the POR stage, the cell maintained a voltage of ≈1 V; after ≈17.8 h, once HO_2_^–^ was fully consumed, the applied potential transitioned to drive the OER. The regenerative system exhibited an overall energy efficiency of 85.9%, with parasitic losses of 14.1%, highlighting robust reproducibility over repeated cycles using the Ni_20_Py catalyst (Figure 8e). Long‐term testing (≈189 h) of the bifunctional Ni_20_Py catalyst on a carbon electrode further confirmed stable HO_2_^–^ production/consumption without voltage degradation, supporting a self‐sustaining system capable of on‐demand hydrogen generation.

#### 2.2.3 Bipolar Peroxide Electrolyzer (BPE)

**Figure 9:**
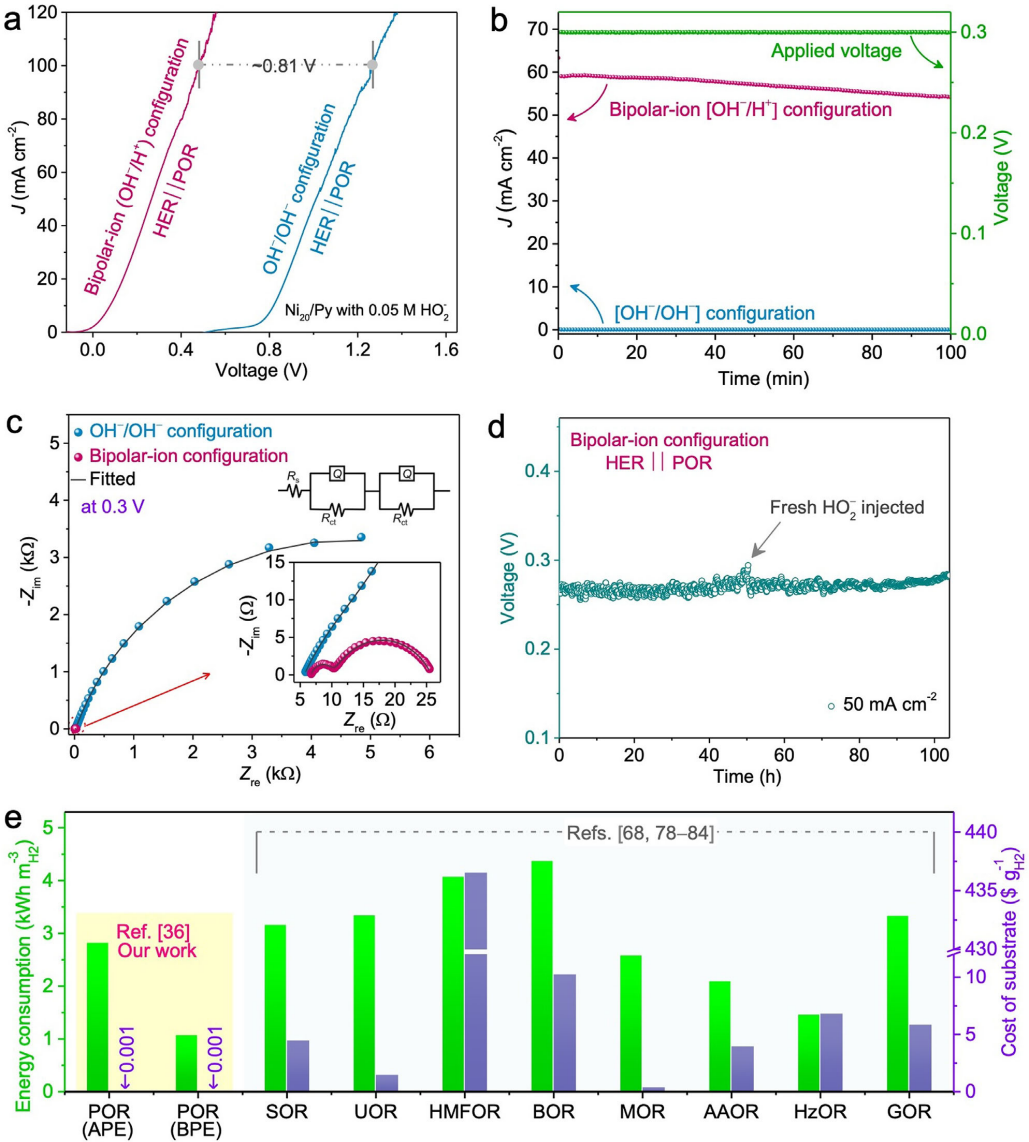
Performance of the bipolar‐ion (OH^–^/H^+^) peroxide electrolyzer (BPE). a) Current density–potential (*J*–*E*) characteristics of the (OH^–^/H^+^) system compared with a symmetric configuration containing 0.05 m HO_2_^–^, recorded at a scan rate of 5 mV s^−1^. b) Current density–time (*J–t*) profiles for both symmetric (OH^–^/H^+^) and asymmetric electrolyzers under a constant driving potential of 0.3 V (right *y*‐axis). In the (OH^–^/H^+^) configuration, the anolyte consisted of pH 14 solution with 0.05 m HO_2_^–^, while the catholyte was maintained at pH 0. c) Nyquist plots from electrochemical impedance spectroscopy (EIS) of symmetric and bipolar‐ion cells at a steady potential of 0.3 V, measured over an AC frequency range of 100 kHz to 10 mHz with a 10 mV perturbation. The inset shows both the equivalent circuit model and showcasing the high‐frequency region. d) Durability assessment (*E–t* profile) of the BPE incorporating the Ni_20_Py catalyst at a constant current density of 50 mA cm^−2^, with periodic injections of 0.5 m HO_2_^–^ into the bipolar electrolyte over 50 h.^[^ ^36^ ^]^ e) Comparative analysis of energy consumption between APE and BPE (operating via POR) against other reported hydrogen‐producing electrolyzers^[^ ^68^, ^78^, ^79^, ^80^, ^81^, ^82^, ^83^, ^84^ ^]^ (green bars, left *y*‐axis), along with estimated feedstock chemical costs (purple bars, right *y*‐axis). ^†^Cost values are adapted from H. Pradhan et al.;^[^ ^83^ ^]^ HO_2_^–^ cost is calculated based solely on the energy required for its synthesis through a 2e^–^ ORR pathway. Reproduced under the terms of the CC‐BY license from reference,^[^ ^36^ ^]^ copyright 2024, published by Wiley–VCH.

The neutralization of OH^–^ and H^+^, which releases usable energy, has been widely investigated as a means of improving diverse energy conversion technologies.^[^ ^73^, ^74^, ^75^, ^76^, ^77^ ^]^ In the case of hydrogen generation, this principle can be implemented through an integrated electrochemical neutralization device that operates sustainably with acidic and alkaline industrial waste streams, effectively providing a near‐zero‐cost electrolyte supply.^[^ ^73^, ^74^, ^75^, ^76^, ^77^ ^]^ This lowers the electrical input required while sustaining or even enhancing current density; as such, we exploited the energy stored in bipolar ions (OH^–^/H^+^) for more efficient hydrogen production. Thermodynamically, up to 0.828 V of electrical energy can be derived from bipolar‐ion gradients at room temperature when applied electrochemically.^[^ ^73^, ^74^, ^75^, ^76^, ^77^ ^]^ Once HO_2_^–^ is introduced into the electrolyte, POR occurs, and the onset potential shifts to lower values, as validated by linear sweep voltammetry of the BPE setup (**Figure** **9**a). Specifically, the BPE with a bipolar‐ion gradient exhibits an onset potential ≈–0.03 V, in contrast to ≈0.77 V for the APE configuration (Figure 9a). Moreover, the BPE system achieves 100 mA cm^−2^ at just 0.48 V, whereas the APE counterpart requires ≈1.29 V under the same conditions (Figure 9a). Even at a low operating voltage of 0.3 V, the BPE delivers high current densities (Figure 9b). Electrochemical impedance spectroscopy at 0.3 V (Figure 9c) further confirms a substantial reduction in charge‐transfer resistance (*R*_ct_) and accelerated kinetics in the BPE relative to the APE system.

The flow‐type BPE device was able to sustain continuous hydrogen generation for more than 100 h at a current density of 50 mA cm^−2^ and an operating voltage of 0.27 V (Figure 9d). In this configuration, the electrolytes consisted of 2 m H_2_SO_4_ as the catholyte and 4 m KOH as the anolyte, supplemented with 0.5 m HO_2_^–^. Periodic HO_2_^–^ injection over 50 h further enhanced the long‐term stability of the system under practical operation. The BPE achieved an impressive overall energy efficiency of 90.7%, highlighting its effectiveness in minimizing the electrical input required for H_2_ production. Energy‐wise, only 1.01 kWh is needed to generate 1 m^3^ of hydrogen, corresponding to an estimated cost of ≈0.03 USD m^−3^ H_2_ when acidic and alkaline waste streams are employed as feedstock.^[^ ^36^ ^]^ Additionally, the cost of HO_2_^–^ synthesis via the two‐electron ORR pathway is extremely low—≈0.034 kWh g^−1^ H_2_, or ≈0.001 USD g^−1^ H_2_.^[^ ^36^ ^]^ In contrast, many of the substrates used in conventional hybrid electrolyzers are derived from nonrenewable sources and carry considerably higher costs (Figure 9e).^[^ ^68^, ^78^, ^79^, ^80^, ^81^, ^82^, ^83^, ^84^ ^]^

Overall, this approach demonstrates that peroxide‐mediated hydrogen generation, enabled by low‐cost, regenerative, and renewable substrates, provides a viable route toward scalable and sustainable hydrogen fuel production. Importantly, it also has the potential to overcome the challenges associated with hydrogen transport and storage infrastructure.

## 3 Conclusion and Future Outlook

This perspective highlights a new class of electrochemical devices enabled by O_2_ redox chemistry based on the reversible 2e^–^ redox couple (O_2_ ⇌ HO_2_^–^), operating at a bifunctional air cathode. The system exhibits excellent catalytic activity and long‐term operational stability during continuous HO_2_^–^ production, achieving a remarkably low energy cost of 152 kJ mol^−1^ while minimizing undesired parasitic PRRs. In GDL mode, the optimized catalyst achieves a superior hydrogen peroxide production rate of 4.5 ± 0.2 mol g_cat_^−1^ h^−1^. In situ studies and theoretical modeling confirm the rapid and reversible formation of NiOOH on the cathode surface during both 2e^–^ ORR and POR processes. This supports highly efficient bifunctional performance within the ZPB configuration. Compared to conventional ZABs, which typically require charging voltages in the range of ≈1.6–2 V, the ZPB operates at much lower charging voltages (1.28–1.48 V). The fast POR kinetics and low onset potentials contribute to improved charge–discharge rates and overall cell durability. This innovative approach achieves a high RTEE, exceeding 95% and delivers over 1000 h performance at a high fixed capacity of 50 mAh cm^−2^ (*j* = 10 mA cm^−2^).

We further extend the 2e^–^ O_2_ redox chemistry into electrolyzer configurations, reducing the theoretical cell voltage from 1.23 V down to as low as –0.06 V. The designed bifunctional catalyst allows hydrogen to be produced directly from HO_2_^–^ generated within the system, removing the necessity for transporting external peroxide. This regenerative, closed‐loop approach lowers substrate expenses dramatically, with costs reduced to ≈0.001 USD g^−1^ H_2_ through the two‐electron ORR route. Additionally, by exploiting bipolar‐ion (OH^−^/H⁺) gradient energy, the system requires nearly three times lower potential to reach high current densities compared with traditional electrolysis. The estimated energy requirement of 1.01 kWh per m^3^ of H_2_ represents a significant improvement in efficiency over most reported systems. Overall, peroxide‐mediated electrochemical energy systems surpass traditional ZABs and earlier peroxide‐based approaches in selectivity, energy efficiency, and durability. This route offers a promising, scalable, and sustainable pathway for H_2_ fuel generation, with the potential to bypass the need for hydrogen transformation, storage, and transportation infrastructure.

## 4 Challenges and Opportunities in Peroxide‐Mediated Redox Chemistry

Despite advances in incorporating peroxide redox chemistry into electrochemical systems, several significant key challenges remain.

### 4.1 Selectivity and Parasitic Reactions

Peroxide‐based systems remain prone to parasitic reactions, especially the further reduction of peroxide via the PRR, a 2e^–^ redox process that is thermodynamically less favorable than the conventional ORR/OER. These side reactions reduce both efficiency and stability, complicating the development of reliable energy storage systems. The key challenge lies in engineering cathodic interfaces that can selectively and reversibly catalyze the O_2_ ⇌ H_2_O_2_ redox couple while suppressing undesired parasitic pathways.

### 4.2 Electrolyte and Electrode Stability in Alkaline Media

In alkaline environments under atmospheric air and high current densities, the 2e^–^ ORR pathway might be disrupted by the formation of insoluble salts such as potassium carbonate (K_2_CO_3_). These precipitates result from the reaction of CO_2_ with the alkaline electrolyte and can block mass transport channels in GDL electrodes. This potential blockage may reduce catalytic activity at the active sites and hinder overall system performance, though we have not detected it in our systems. Advancing bifunctional catalyst performance in near‐neutral media may help maintain selective, reversible 2e^–^ ORR/POR behavior while reducing carbonate fouling and mitigating the utilized corrosive media.

### 4.3 Peroxide Stability and System Degradation

High concentrations of peroxide, especially under practical operating current densities, pose several risks. These include self‐decomposition of peroxide, anode corrosion, membrane degradation, and peroxide crossover between battery compartments—all of which compromise lifetime, efficiency, and reversibility. Innovations in cell architecture, chemically robust membranes, and stabilizing additives are essential to mitigate peroxide reactivity in concentrated states.

Beyond zinc‐based systems, the peroxide‐mediated approach can be extended to other high‐energy‐density metal–peroxide chemistries, including magnesium–hydrogen peroxide (Mg–H_2_O_2_), aluminum–hydrogen peroxide (Al–H_2_O_2_), and iron–hydrogen peroxide (Fe–H_2_O_2_) systems. Each brings unique opportunities and engineering challenges for achieving safe, scalable, and high‐performance storage and conversion.

In summary, peroxide‐mediated pathways offer a powerful alternative to conventional 4e^–^ O_2_ redox chemistry. By integrating advanced catalysts, interface engineering, and optimized architectures, this strategy could deliver energy‐efficient, scalable, and commercially viable solutions for next‐generation electrochemical energy technologies.

## Conflict of Interest

The authors declare no conflict of interest.
